# Preparation and Characterization of Nanoliposomes Entrapping Medium-Chain Fatty Acids and Vitamin C by Lyophilization

**DOI:** 10.3390/ijms141019763

**Published:** 2013-09-30

**Authors:** Shuibing Yang, Chengmei Liu, Wei Liu, Haixia Yu, Huijuan Zheng, Wei Zhou, Yaqin Hu

**Affiliations:** 1 Department of Food Science and Nutrition, Zhejiang Key Laboratory for Agro-Food Processing, Fuli Institute of Food Science, Zhejiang University, Hangzhou 310058, China; E-Mail: shuibingyang1984@aliyun.com; 2 Ocean Research Center of Zhoushan, Zhejiang University, Zhoushan 316021, China; E-Mail: haixiahome@gmail.com; 3 State Key Laboratory of Food Science and Technology, Department of Life Science and Food Engineering, Nanchang University, Nanchang 330047, China; E-Mails: liuchengmei@aliyun.com (C.L.); liuwei@ncu.edu.cn (W.L.); huijuanzheng87@gmail.com (H.Z.); ncuskzhouwei@163.com (W.Z.)

**Keywords:** medium-chain fatty acids, vitamin C, double emulsion, dynamic high pressure microfluidization, lyophilization

## Abstract

The complex nanoliposomes encapsulating both a hydrophilic drug vitamin C (vit C) and hydrophobic drug medium-chain fatty acids (MCFAs) was prepared by combining double emulsion method with dynamic high pressure microfluidization. The complex nanoliposomes was further freeze-dried under −86 °C for 48 h with sucrose at the sucrose/lipids ratio of 2:1(*w*/*w*) in order to enhance its stability. The freeze-dried complex nanoliposomes under the suitable conditions exhibited high entrapment efficiency of MCFAs (44.26 ± 3.34)%, relatively high entrapment efficiency of vit C (62.25 ± 3.43)%, low average size diameter (110.4 ± 7.28) nm and good storage stability at 4 °C for 60 days with slight changes in mean particle diameter and drug entrapment efficiencies. The results of transmission electron microscopy of freeze-dried complex nanoliposomes also showed that the freeze-dried samples with sucrose were stable without great increase in their particle sizes and without destroying their spherical shape. The results indicated that sucrose presented well protection effects in MCFAs-vit C complex nanoliposomes, suggesting the possibility of further usage in commercial liposomes.

## 1. Introduction

Hydrophobic drug medium-chain fatty acids (MCFAs) composed of octanoic acid and decanoic acid has unique characteristics different from long-chain fatty acids [1]. MCFAs are more rapidly hydrolyzed into triglycerides by pancreatic lipase than long chain fatty acids, and transported via portal vein to the liver where they are preferentially β-oxidized by carnitine-independent pathway [2]. In addition, they can save the amount of protein [1], inhibit the formation of body fat and suppress diseases caused by pathogenic bacteria [3,4]. However, the water solubility of MCFAs is relatively weak, which often prevents their parenteral and oral administration from being effective. In addition, an excess of non-esterified MCFAs may have serious side effects such as stimulation to the secretion of cholecystokinin and perhaps other intestinal hormones [5]. As one kind of typical water-soluble nutrient, vitamin C (vit C) is essential for humans and other certain animal species [6]. It can reduce the damage of free radicals on the skin, delay aging and reduce the formation of melanin [7–9]. However, vit C could be easily oxidized under ambient conditions and light exposure. This could cause an obvious decrease of its utilization [10].

Suitable drug carriers are needed as the delivery system in order to protect them and enhance their bioavailability. It is very challenging to entrap the two drugs in a drug carrier, due to their quite different properties of drugs. An attractive method for entrapping water-soluble and insoluble drugs is the use of liposome [11]. The phospholipids bilayer membrane structure of liposome guarantees that hydrophilic and hydrophobic drugs can be efficiently and simultaneously entrapped in it [12]. The liposome can prolong the residence time of the drugs in the blood, making the drugs release slowly *in vivo* [13]. Due to better distributions in the organisms, small nanoliposomes with diameters of about 100 nm are frequently used as a delivery system for drugs [14]. The bioavailability of encapsulated components is higher in nanoliposomes than in conventional liposomes [15,16]. Preparation of complex nanoliposomes is very challenging by traditional methods, due to the difficulties in the implementation of small particle size and high entrapment efficiency.

Many methods were used for preparing liposomes, most of which exhibit favorable characteristics when entrapping one hydrophobic or hydrophilic drug [17,18]. However, those methods had certain disadvantages when preparing complex liposomes that entrap both hydrophobic and hydrophilic drugs. The problems limiting the manufacture and development of liposomes had been stability issues, batch to batch reproducibility, low drug entrapment and particle size control [19]. Thus, combined methods consisting of two or more different methods are investigated for preparation of liposomes that entrap both hydrophobic and hydrophilic drugs. Currently, double emulsion was reported to be the most appropriate method to encapsulate hydrophilic drugs [20,21].

Dynamic high pressure microfluidization (DHPM) is a technology that uses the combined forces of shear, cavitation, and ultra-high pressures up to 200 MPa [22]. It can reduce the particle sizes of liposomes greatly and could be applied for large-scale production of liposomes in a continuous process without employing toxic solvents [23–26]. In this study, traditional methods and DHPM were combined for preparing MCFAs-vit C complex nanoliposomes suspension. However, ordinary liposomes exist in liquid form, which leads to several unstable problems, such as particle aggregation sedimentation and drug leakage [19]. After liposomes were freeze-dried, these problems could be avoided [27]. While fusion of phospholipids membranes may also occur during the freezing, drying and rehydration, the addition of lyoprotectants such as sucrose, trehalose, or lactose is necessary for preventing leakage or fusion [28–30]. Up to now, no report about freeze-dried MCFA-vit C nanoliposomes has been found. In order to enhance the stability of liposomes, strengthen its functional effect, the lyophilized liposomes were prepared in this study with an expectation on its application in feedstuff or as injection and aerosol after rehydration.

## 2. Results and Discussion

### 2.1. Characteristics of Freeze-Dried MCFAs-Vit C Complex Nanoliposomes

The changes of particle size and drug entrapment efficiencies of the liposomes before and after lyophilization are important for evaluating the effects of freeze-drying process on liposomes. The characteristics of the freeze-dried complex nanoliposomes in the presence or absence of sucrose compared with liposomes suspension before lyophilization were shown in Table 1. The results showed that the qualities of MCFAs-vit C complex nanoliposomes improved after the addition of sucrose, which made freeze-dried liposomes with relative small size increase and high drug entrapment efficiencies compared with liposomes suspension before lyophilization. The freeze-dried complex nanoliposomes without any cryoprotectant (the control group) exhibited a larger mean particle diameter of (151.4 ± 10.76) nm, and drugs entrapment efficiencies could not be detected because of the damages caused by lyophilization. Similar results were found in our previous report [31], which indicated that the freezing-drying process has a small effect on the liposome size, resulting in a slight increase in average diameter from 250.1 to 263.3 nm. On the other hand, formulations prepared without saccharose as cryoprotectant presented an increase in vesicle size after lyophilization. It can be concluded that sucrose succeeded in avoiding size increase and preventing drug leakages.

### 2.2. Transmission Electron Microscopy of Complex Nanoliposomes

**Figure 1:**
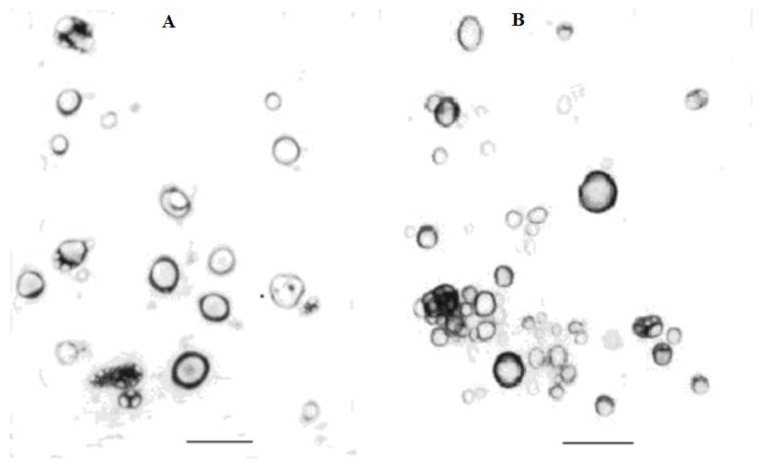
TEM profile of liposomes. (**A**) Profile of complex liposomes suspension; (**B**) Profile of freeze-dried complex liposomes. The bar in the figures represents 400 nm. The amplification time was 25 × 10^3^.

The morphology of the complex nanoliposomes under the optimum conditions was evaluated by transmission electron microscopy (TEM) experiments. The typical micrographs of the freshly prepared liposome suspension and the freeze-dried liposomes with sucrose as the suitable cytoprotectant are shown respectively in Figure 1A,B. It was found that both freshly prepared liposomes and freeze-dried liposomes were unilamellar in nature. These liposomes could easily be identified as discrete particles that were predominantly spherical or elliptic in shape. Microscopic observations of liposomes suspension revealed few aggregated or semifused vesicles, and majority of lipoplexes are in the form of individual vesicles. While, freeze-dried complex liposomes presented partly aggregated or semifused vesicles, which may be concerned with the complex process of the lyophilization or the shrinking during drying on the EM grid. The sizes of liposomes observed in the typical micrographs were smaller than the results obtained by particle size measurements. This is probably due to hydration and swelling of the particles in aqueous buffer [32]. The dissimilarities between the TEM and the particle size measurements of the liposomes could be explained by considering that the samples in the TEM investigations were prepared by allowing the solvent to evaporate [33]. The results of TEM indicated that the freeze-dried samples were stable without great increase in their particle sizes and without destroying their spherical shape. These all suggested that the cytoprotectant sucrose was successful in protecting the complex nanoliposomes.

### 2.3. Storage Stability

The stability of freshly prepared and freeze-dried MCFAs-vit C complex liposomes by DE-DHPM stored from light at 4 °C is shown in Table 2. The particle size of freshly prepared liposomes had minor changes within 60 days, from (99.5 ± 2.08) nm to (132.3 ± 5.26) nm. The increment of a 33% in size after 60 days was possibly due to the partial aggregation brought by the minimization of high surface-to-volume ratios. And just the increment of a 36% in size after 60 days was observed for freeze-dried complex liposomes. In our previous studies, the stability of MCFAs liposomes prepared by DHPM at 4 °C for 3 months was investigated, which presented good stability with relative bigger changes in particle size from (86.8 ± 1.01) nm to (172.6 ± 5.51) nm [34]. In addition, freeze-dried MCFAs-vit C complex nanoliposomes experienced little loss of entrapped MCFAs and vit C during 60 days. These results showed that the MCFAs-vit C complex liposomes prepared had a favorable stability during storage, suggesting that freeze-drying with certain amount of sucrose had excellent effects for long-term storage of liposomal formulation.

## 3. Experimental Section

### 3.1. Materials

Medium-chain fatty acids were kindly provided by a USA company (UPMC, Pittsburgh, PA, USA). Octanoic acid methyl ester and decanoic acid methyl ester were purchased from AccuStandard (New Haven, CT, USA). Vitamin C was purchased from Sinopharm Chemical Reagent Co. Ltd. (Shanghai, China). Soybean phosphatidylcholine (SPC) was provided by Merya’s Lecithin Co. Ltd. (Beijing, China). Cholesterol (CHO) was obtained from Tianjin Damao Chemical Reagent Co. Ltd. (Tianjin, China). *N*-hexane and methanol were of chromatographic grade. Acetic acid, EDTA, fast blue salt B, sucrose and other reagent chemicals were all of analytical grade.

### 3.2. Preparation of MCFAs-Vit C Complex Nanoliposomes by DE-DHPM

MCFAs-vit C complex liposomes were prepared by double emulsion (DE) [35] under the optimum conditions as follows. A 100:25:4 mass ratio of soybean phosphatidylcholine (1200 mg), cholesterol (300 mg) and vitamin E (48 mg) were dissolved in 15 mL absolute ethanol containing MCFAs (300 mg), followed by injection of 2 mL twice-distilled water under vigorous stirring at a temperature of 50 °C. The primary emulsion was placed in a rotary evaporator (RE52-02, Yarong Instrument Co., Shanghai, China) to remove part of the solvent under reduced pressure of 0.08 MPa for 10 min. 25 mL twice-distilled water containing 450 mg surfactant tween-80 (Tianjin Damao Chemical Reagent Co. Ltd, Tianjin, China) and 60 mg vit C (Sinopharm Chemical Reagent Co. Ltd, Shanghai, China) was added. The resulting emulsion was constantly agitated for about 20 min at 50 °C, and any traces of the solvent were removed by rotary evaporation under reduced pressure at 50 °C for about 1 h until it formed a milk-like liquid, resulting in the formation of crude complex liposomes. To prepare MCFAs-vit C complex nanoliposomes, the crude liposomes were further treated by a microfluidizer (M-110EH30, Microfluidic Corporation, Newton, MA, USA) under optimum treatment condition (120 MPa, three passes) at room temperature.

### 3.3. Preparation of Freeze-Dried MCFAs-Vit C Complex Nanoliposomes

The complex nanoliposomes suspensions prepared by DE-DHPM under the optimal conditions were filtered by the 0.45 μm Millipore filter, and then various amounts of sucrose was added to the liposomes suspensions to provide the desired mass ratios of saccharide and lipids with total soybean phosphatidylcholine at the concentration of 2% (*w*/*v*). Mean particle size and drug entrapment efficiencies of the complex nanoliposomes after rehydration were adopted as the main indexes. Sucrose was used in the complex nanoliposomes with cryoprotectant and phosphatidylcholine at the mass ratio of 2:1 (*w*/*w*).

In deep-freeze equipment, the nanoliposomes suspensions were pre-freezed for 6 h to a terminal temperature of −80 °C, and then dried at −86 °C with a vacuity of 0.1 mbar for 48 h in the vacuum freeze drying plant. The freeze-dried products of MCFAs-vit C complex nanoliposomes were henceforth obtained.

### 3.4. Reconstitution of the Freeze-Dried MCFAs-Vit C Complex Nanoliposomes

Aqueous suspensions were immediately formed upon the rehydration of the lyophilized products with distilled water to the original volume, and the ratio of the freeze-dried products to distilled water was 1:50–1:100 [36].

### 3.5. Determination of Entrapment Efficiency (EE) of MCFAs

Entrapment efficiency (*EE*) of MCFAs was determined according to the methods of our previous studies [34]. About 1 mL of MCFAs-vit C complex nanoliposomes was centrifuged at 12000 × *g* for 20 min, and 2 mL of *n*-hexane was added to withdraw unentrapped MCFAs. The rest of the suspension was mixed with 7 mL methanol, and then sonicated for 25 min to demulsify the complex nanoliposomes. The fatty acid methyl esters (FAMEs) were made by direct transesterification [37,38]. FAMEs were measured by gas chromatography (Agilent 6890 Series GC System, Aglient Technologies, Santa Clara, CA, USA) with a flame ionization detector. The chromatographic column was HP-innowax polyethylene glycol (30 m × 0.32 mm × 0.5 μm) and the flow rates of various gases were nitrogen, 20 mL/min; hydrogen, 40 mL/min; air, 450 mL/min. The temperatures of injection port and detector were maintained at 280 °C. The oven temperature was programmed to maintain at 150 °C for 5 min, then rise to 180 °C at a rate of 6 °C/min and stay at 180 °C for 2 min. The injection volume was 1 μL. Peak areas and retention times were calculated and FAMEs were identified by comparing retention times to the standard octanoic acid methyl ester and decanoic acid methyl ester. The entrapment efficiency of MCFAs (EE_MCFAs_) was calculated from Equation (1):

(1) $$EE_{MCFAs}=\left(W_{en}/W_{total}\right)\times 100\%$$

where *W*_en_ is the analyzed weight of MCFAs encapsulated in the nanoliposomes, and *W*_total_ is the initial weight of MCFAs added.

### 3.6. Determination of Encapsulation Efficiency (EE) of Vit C

Non-encapsulated vit C was separated from MCFAs-vit C complex nanoliposomes by centrifugation. About 5 mL of liposomes were centrifuged at 12000 × *g* for 20 min. Then, the drug content in the supernatant was quantified by UV-Visible spectrophotometry [39]. 1 mL of above treated mixture solution was placed in a 10 mL colorimetric tube followed by adding 0.3 mL of EDTA (0.25 M), 0.5 mL of acetic acid (0.5 M) and 1.25 mL of fast blue salt B (2 g/L) in sequence, then diluted to 10 mL with deionized water. 20 min later, the above mixture was determined spectrophotometrically at 420 nm using a UV-Visible spectrophotometer (T6, Purkinje General, Beijing, China). The entrapment efficiency of vit C (EE_Vit C_) was calculated from Equation (2):

(2) $$EE_{vit.C}=\left[1-\left(W_{free}/W_{total}\right)\right]\times 100\%$$

where *W*_free_ is the analyzed weight of free vit C, and *W*_total_ is the initial weight of vit C added in the preparation.

### 3.7. Characteristics of MCFAs-Vit C Complex Nanoliposomes

#### 3.7.1. Particle Size and Size Distribution

The particle sizes of MCFAs-vit C complex nanoliposomes prepared by DE-DHPM were determined by dynamic laser light scattering method at 25 °C using a Nicomp 380 ZLS (Santa Barbara, CA, USA). The intensity was detected at an angle of 90°. MCFAs-vit C complex liposomes were diluted with twice-distilled water before measurement [40].

#### 3.7.2. Transmission Electron Microscopy (TEM)

MCFAs-vit C complex liposome was diluted approximately at 1:10 with twice-distilled water. One drop of the diluted sample was left alone for 3 min. The solution was placed on a copper grid for 5 min before the excess liquid was sipped up by the filter papers [41,42], and then air-dried at room temperature before being observed under TEM (Hitachi H-600, Tokyo, Japan).

#### 3.7.3. Stability of MCFAs-Vit C Complex Nanoliposomes

The stability was assessed by comparing different changes in mean diameters and drug encapsulation efficiencies of freshly prepared and freeze-dried MCFAs-vit C complex nanoliposomes prepared by DE-DHPM at fixed time intervals (1, 4, 7, 10, 13, 16, 30, 45, 60 days) respectively. The complex nanoliposomes were stored from light at 4 °C in a sealed condition [43].

### 3.8. Statistical Analysis

All experiments were done in triplicate unless otherwise specified and the values were expressed as means ± standard deviation (SD) for three different experiments. Data were subjected to statistical analysis by Student’s *t*-test.

## 4. Conclusions

MCFAs-vit C complex nanoliposomes suspensions were prepared by DE-DHPM. In order to enhance the stability of MCFA and vit C, the lyophilized complex nanoliposomes were prepared. After being freeze-dried under the optimum conditions of −86 °C for 48 h, freeze-dried MCFAs-vit C complex nanoliposomes with sucrose as the cryoprotectant at a sucrose/lipids ratio of 2:1 (*w*/*w*) had a good size distribution, good appearance and relatively high entrapment efficiency of MCFAs and vit C. The MCFAs entrapment efficiency of nanoliposomes was (44.26 ± 3.34)% with the mean diameter of (110.4 ± 7.28) nm, while vit C was (62.25 ± 3.43)%. MCFAs-vit C complex nanoliposomes exhibited a favorable stability during the storage period of 60 days at 4 °C. The TEM results of MCFAs-vit C complex nanoliposomes indicated that the freeze-dried samples with sucrose were stable without great changes in their characters. In conclusion, freeze-drying with sucrose was proved to be a successful method to form liposomal for long-term storage.

## Figures and Tables

**Table 1:** The effects of freeze-drying and sucrose on the characters of the complex medium-chain fatty acids (MCFAs)-vit C nanoliposomes.

Samples	Particle size (nm)	Polydispersity index	*EE*_vit C_ (%)	*EE*_MCFAs_ (%)
Sample before lyophilization	115.3 ± 9.37	0.257	61.37 ± 3.17	46.23 ± 1.25
Lyophilized nanoliposomes with sucrose	110.4 ± 7.28	0.232	62.25 ± 3.43	44.26 ± 3.34
Lyophilized nanoliposomes without sucrose	151.4 ± 10.76	0.384	-	-

“-” in the table represents “the value can’t be detected”.

**Table 2:** Characteristics of MCFAs-vit C complex nanoliposomes during storage at 4 °C.

Days	Mean diameter (nm)		Polydispersity index		*EE*_MCFAs_ (%)		*EE*_vit C_ (%)	

	FPL	FDL	FPL	FDL	FPL	FDL	FPL	FDL
1	99.5 ± 2.08	110.4 ± 7.28	0.232	0.257	46.83 ± 2.98	44.26 ± 3.34	65.32 ± 3.42	62.25 ± 3.43
4	98.8 ± 4.68	112.8 ± 6.62	0.267	0.273	45.54 ± 3.14	43.32 ± 5.24	64.82 ± 2.68	63.72 ± 3.57
7	108 ± 3.87	118 ± 5.23	0.343	0.316	42.63 ± 2.55	42.27 ± 4.23	63.77 ± 2.42	61.25 ± 4.42
10	112 ± 2.38	125 ± 4.25	0.310	0.328	44.01 ± 3.25	41.01 ± 4.55	61.57 ± 1.88	59.27 ± 3.82
13	118 ± 4.98	130 ± 4.36	0.384	0.354	40.75 ± 2.28	38.24 ± 5.16	62.76 ± 1.75	57.24 ± 4.36
16	126 ± 3.96	138 ± 5.22	0.331	0.315	40.91 ± 3.68	37.15 ± 6.53	61.43 ± 2.04	55.32 ± 3.75
30	129 ± 5.17	140 ± 4.13	0.375	0.363	42.75 ± 2.87	37.52 ± 5.32	60.23 ± 2.68	55.15 ± 4.59
45	130 ± 4.21	143 ± 5.15	0.325	0.343	42.06 ± 1.86	36.21 ± 3.15	59.75 ± 4.52	53.25 ± 4.23
60	132.3 ± 5.26	150.2 ± 4.26	0.370	0.389	40.39 ± 3.17	38.27 ± 4.65	58.98 ± 3.08	52.98 ± 5.05

Note: “FPL” in the table refers to “Freshly prepared liposome”; “FDL” in the table refers to “Freeze-dried liposome”.

## References

[1] Jones P.M., Butt Y.M., Bennett M.J. (2004). Effects of odd-numbered medium-chain fatty acids on the accumulation of long-chain 3-hydroxy-fatty acids in long-chain l-3-hydroxyacyl CoA dehydrogenase and mitochondrial trifunctional protein deficient skin fibroblasts. Mol. Genet. Metab.

[2] Bach A.C., Babayan V.K. (1982). Medium-chain triglycerides: An update. Am. J. Clin. Nutr.

[3] Hirazawa N., Oshima S., Hara T., Mitsuboshi T., Hata K (2001). Antiparasitic effect of medium-chain fatty acids against the ciliate *Cryptocaryon irritans* infestation in the red sea bream *Pagrus major*. Aquaculture.

[4] Wong P.Y.Y., Nakamura S., Kitts D.D. (2006). Functional and biological activities of casein glycomacropeptide as influenced by lipophilization with medium and long chain fatty acid. Food Chem.

[5] Decuypere J.A., Dierick N.A. (2003). The combined use of triacylglycerols containing medium-chain fatty acids and exogenous lipolytic enzymes as an alternative to in-feed antibiotics in piglets: Concept, possibilities and limitations. An overview. Nutr. Res. Rev.

[6] Liu N., Park H.J. (2010). Factors effect on the loading efficiency of Vitamin C loaded chitosan-coated nanoliposomes. Colloids Surf. B.

[7] Tejero E., Perichart O., Pfeffer F., Casanueva E., Vadillo-Orteg F (2003). Collagen synthesis during pregnancy, vitamin C availability, and risk of premature rupture of fetal membranes. Int. J. Gynecol. Obstet.

[8] Wintergerst E.S., Maggini S., Hornig D.H. (2006). Immune-enhancing role of vitamin C and zinc and effect on clinical conditions. Ann. Nutr. Metab.

[9] Lin J., Selim M.A., Shea C.R., Grichnik J.M., Omar M.M., Monteiro-Riviere N.A., Pinnell S.R. (2003). UV photoprotection by combination topical antioxidants vitamin C and vitamin E. J. Am. Acad. Dermatol.

[10] Zhang L., Lerner S., Rustrum W.V., Hofmann G.A. (1999). Electroporation-mediated topical delivery of vitamin C for cosmetic applications. Bioelectrochem. Bioenerg.

[11] Kirjavainen M., Urtti A., Jaaskelainen I., Suhonen T.M., Paronen P., Valjakka-Koskela R., Kiesvaara J., Monkkonen J. (1996). Interaction of liposomes with human skin *in vitro*-the influence of lipid composition and structure. Biochim. Biophys. Acta.

[12] Ma Q., Kuang Y., Hao X (2009). Preparation and characterization of tea polyphenols and vitamin E loaded nanoscale complex liposome. J. Nanosci. Nanotechnol.

[13] Lee J.S., Chung D., Lee H.G. (2008). Preparation and characterization of calcium pectinate gel beads entrapping catechin-loaded liposomes. Int. J. Biol. Macromol.

[14] Kaiser J.M., Imai H., Haakenson J.K., Brucklacher R.M., Fox T.E., Shanmugavelandy S.S., Unrath K.A., Pedersen M.M., Dai P., Freeman W.M. (2013). Nanoliposomal minocycline for ocular drug delivery. Nanomedicine.

[15] Acosta E (2009). Bioavailability of nanoparticles in nutrient and nutraceutical delivery. Curr. Opin. Colloid Interface Sci.

[16] Huang Q.R., Yu H.L., Ru Q.M. (2010). Bioavailability and delivery of nutraceuticals using nanotechnology. J. Food Sci.

[17] Xia S., Xu S (2005). Ferrous sulfate liposomes: Preparation, stability and application in fluid milk. Food Res. Int.

[18] Marsanasco M., Marquez A.L., Wagner J.R., del Valle Alonso S., Chiaramoni N.S. (2011). Liposomes as vehicles for vitamins E and C: An alternative to fortify orange juice and offer vitamin C protection after heat treatment. Food Res. Int.

[19] Sharma A., Sharma U.S. (1997). Liposomes in drug delivery: Progress and limitations. Int. J. Pharm.

[20] Alex R., Bodmeier R (1990). Encapsulation of water-soluble drugs by a modified solvent evaporation method. I. Effect of process and formulation variables on drug entrapment. J. Microencapsul.

[21] Cohen S., Yoshioka T., Lucarelli M., Hwang L.H., Langer R (1991). Controlled delivery systems for proteins based on poly (lactic/glycolic acid) microspheres. Pharm. Res.

[22] Liu W., Liu J., Xie M., Liu C., Liu W., Wan J (2009). Characterization and high-pressure microfluidization-induced activation of polyphenoloxidase from Chinese Pear (Pyrus pyrifolia Nakai). J. Agric. Food Chem.

[23] Takahashi M., Inafuku K., Miyagi T., Oku H., Wada K., Imura T., Kitamoto D (2007). Efficient preparation of liposomes encapsulating food materials using lecithins by a mechanochemical method. J. Oleo Sci.

[24] Barnadas-Rodriguez R., Sabes M (2001). Factors involved in the production of liposomes with a high-pressure homogenizer. Int. J. Pharm.

[25] Zheng S., Alkan-Onyuksel H., Beissinger R.L., Wasan D.T. (1999). Liposome microencapsulations without using any organic solvent. J. Dispers. Sci. Technol.

[26] Jafari S.M., He Y., Bhandar B (2006). Nano-emulsion production by sonication and microfluidization—A comparison. Int. J. Food Prop.

[27] Cui J.X., Li C.L., Deng Y.J., Wang Y., Wang W (2006). Freeze-drying of liposomes using tertiary butyl alcohol/water cosolvent systems. Int. J. Pharm.

[28] Tang X., Pikal M (2003). Design of freeze-drying processes for pharmaceuticals: Practical advice. Pharm. Res.

[29] Komatsu H., Saito H., Okada S., Tanaka M., Egashira M., Handa T (2001). Effects of the acyl chain composition of phosphatidylcholines on the stability of freeze-dried small liposomes in the presence of maltose. Chem. Phys. Lipids.

[30] Alexopoulou E., Georgopoulos A., Kagkadis K.A., Demetzos C (2006). Preparation and characterization of lyophilized liposomes with incorporated quercetin. J. Liposome Res.

[31] Liu C.M., Yang S.B., Liu W., Wang R.L., Wan J., Liu W (2010). Preparation and characterization of medium-chain fatty acid liposomes by lyophilization. J. Liposome Res.

[32] Bharali D.J., Sahoo S.K., Mozumdar S., Maitra A (2003). Cross-linked polyvinylpyrrolidone nanoparticles: A potential carrier for hydrophilic drugs. J. Colloid Interface Sci.

[33] Angelini G., Boncompagni S., de Maria P., de Nardi M., Fontana A., Gasbarri C., Menna E (2007). Layer-by-layer deposition of shortened nanotubes or polyethylene glycol-derivatized nanotubes on liposomes: A tool for increasing liposome stability. Carbon.

[34] Liu W., Liu W.L., Liu C.M., Liu J.H., Yang S.B., Zheng H.J., Lei H.W., Ruan R., Li T., Tu Z.C. (2011). Medium-chain fatty acid nanoliposomes for easy energy supply. Nutrition.

[35] Hombreiro Perez M., Zinutti C., Lamprecht A., Ubrich N., Astier A., Hoffman M., Bodmeier R., Maincent P (2000). The preparation and evaluation of poly ([epsilon]-caprolactone) microparticles containing both a lipophilic and a hydrophilic drug. J. Control. Release.

[36] Wang T., Deng Y.J., Geng Y.H., Gao Z., Zou J., Wang Z (2006). Preparation of submicron unilamellar liposomes by freeze-drying double emulsions. Biochim. Biophys. Acta.

[37] Lepage G., Roy C.C. (1986). Direct transesterification of all classes of lipids in a one-step reaction. J. Lipid Res.

[38] Samman S., Chow J.W.Y., Foster M.J., Ahmad Z.I., Phuyal J.L., Petocz P (2008). Fatty acid composition of edible oils derived from certified organic and conventional agricultural methods. Food Chem.

[39] Hernandez Y., Lobo M.G., Gonzalez M (2006). Determination of vitamin C in tropical fruits: A comparative evaluation of methods. Food Chem.

[40] Zhao L., Xiong H., Peng H., Wang Q., Han D., Bai C., Liu Y., Shi S., Deng B (2011). PEG-coated lyophilized proliposomes: Preparation, characterizations and *in vitro* release evaluation of vitamin E. Eur. Food Res. Technol.

[41] Alonso-Romanowski S., Chiaramoni N.S., Lioy V.S., Gargini R.A., Viera L.I., Taira M.C. (2003). Characterization of diacetylenic liposomes as carriers for oral vaccines. Chem. Phys. Lipids.

[42] Hatziantoniou S., Nezis I.P., Margaritis L.H., Demetzos C (2007). Visualisation of liposomes prepared from skin and stratum corneum lipids by transmission electron microscopy. Micron.

[43] Christensen D., Foged C., Rosenkrands I., Nielsen H.M., Andersen P., Agger E (2007). Trehalose preserves DDA/TDB liposomes and their adjuvant effect during freeze-drying. Biochim. Biophys. Acta.

